# Morphological and functional changes in the vertebral column with increasing aquatic adaptation in crocodylomorphs

**DOI:** 10.1098/rsos.150439

**Published:** 2015-11-04

**Authors:** Julia L. Molnar, Stephanie E. Pierce, Bhart-Anjan S. Bhullar, Alan H. Turner, John R. Hutchinson

**Affiliations:** 1 Department of Anatomy, Howard University College of Medicine, Washington, DC 20059, USA; 2 Museum of Comparative Zoology and Department of Organismic and Evolutionary Biology, Harvard University, Cambridge, MA 02138, USA; 3 Department of Geology and Geophysics, Yale University, New Haven, CT 06520, USA; 4 Department of Anatomical Sciences, Stonybrook University, Stony Brook, NY 11794, USA; 5 Structure and Motion Lab, Department of Comparative Biomedical Sciences, The Royal Veterinary College, Hawkshead Lane, Hatfield, Hertfordshire AL9 7TA, UK

**Keywords:** crocodylomorph, vertebrae, axial skeleton, stiffness, range of motion, aquatic adaptation

## Abstract

The lineage leading to modern Crocodylia has undergone dramatic evolutionary changes in morphology, ecology and locomotion over the past 200+ Myr. These functional innovations may be explained in part by morphological changes in the axial skeleton, which is an integral part of the vertebrate locomotor system. Our objective was to estimate changes in osteological range of motion (RoM) and intervertebral joint stiffness of thoracic and lumbar vertebrae with increasing aquatic adaptation in crocodylomorphs. Using three-dimensional virtual models and morphometrics, we compared the modern crocodile *Crocodylus* to five extinct crocodylomorphs: *Terrestrisuchus*, *Protosuchus*, *Pelagosaurus*, *Steneosaurus* and *Metriorhynchus*, which span the spectrum from terrestrial to fully aquatic. In *Crocodylus*, we also experimentally measured changes in trunk flexibility with sequential removal of osteoderms and soft tissues. Our results for the more aquatic species matched our predictions fairly well, but those for the more terrestrial early crocodylomorphs did not. A likely explanation for this lack of correspondence is the influence of other axial structures, particularly the rigid series of dorsal osteoderms in early crocodylomorphs. The most important structures for determining RoM and stiffness of the trunk in *Crocodylus* were different in dorsoventral versus mediolateral bending, suggesting that changes in osteoderm and rib morphology over crocodylomorph evolution would have affected movements in some directions more than others.

## 1. Introduction

Crocodiles, alligators and gharials are the only extant representatives of the clade Crocodylomorpha, whose members span the continuum from terrestrial to aquatic. Early crocodylomorphs had more erect (as opposed to sprawling) limb postures and parasagittal gaits and were probably highly terrestrial, like many other archosaurs [1], whereas later crocodylomorphs diversified to inhabit both terrestrial and aquatic environments [2]. One extinct clade, Thalattosuchia, includes obligatory aquatic marine specialists. Modern crocodylians are semi-aquatic and use a range of postures and gaits between sprawling and erect; therefore, the generally more sprawling posture of modern crocodylians must have been re-acquired at some point in their evolution. One unusual attribute of the crocodylomorph lineage is the convergent evolution of erect postures and asymmetrical (bounding and galloping) gaits otherwise found only in mammals. Only small (shorter than 3 m) crocodylids and gavialids (not alligatorids) have been observed to use asymmetrical gaits; architectural properties of the limb muscles may be a limiting factor [3]. Bounding and galloping abilities in modern crocodylians may have been inherited from their more terrestrial ancestors [1], or these abilities may have originated more recently [4].

In order to understand when and how crocodylians acquired their locomotor abilities, we must reconstruct the locomotor characteristics of extinct members of the crocodylomorph lineage. It is the existence of modern crocodylians that makes Crocodylomorpha such an attractive group in which to study the locomotor evolution of vertebrates. Methods for predicting locomotion in extinct animals should, ideally, be validated in closely related extant taxa [5,6], but this is not easy to do in groups whose extant members have very different locomotor specializations from their extinct ancestors, such as non-avian dinosaurs and birds. Crocodylomorpha provides a rare opportunity to test hypotheses about form–function relationships in a group outside Mammalia whose members once filled a wide variety of ecological niches and whose extant members are capable of diverse locomotor modes (e.g. various symmetrical and asymmetrical gaits), some of which might be ancient holdovers from more terrestrial ancestors. Furthermore, current hypotheses about locomotion in extinct crocodylomorphs are supported by multiple lines of evidence, including limb proportions and joint morphology (e.g. [1,7–10]), palaeobiogeography [1] and trackways [11] (see Discussion for details).

Because the vertebral column is an important part of the locomotor system, its morphology can give essential clues about locomotion in extinct vertebrates. Studies of extant animals have shown that biomechanical properties and, to some extent, locomotor habits can be predicted from vertebral morphology, provided differences among species are taken into account [12–19]. However, few studies thus far have sought to test or apply these principles in non-mammalian tetrapods. Recently, we [20] identified several morphometric parameters that could predict intervertebral joint (IVJ) passive stiffness in modern crocodiles and described the relationship among stiffness, range of motion (RoM) and axial tissues. We also inferred that the role of the axial column in crocodylian locomotion may be functionally different from that in mammals, even during analogous gaits.

Based on analogy with mechanical structures, Salisbury & Frey [21] defined several categories of axial morphology in extant and extinct crocodylomorphs and predicted how each would have functioned during locomotion. According to that study, modern crocodylians accommodate locomotor forces on the vertebral column primarily with their procoelous (each centrum bearing a cranial socket or cotyle and a caudal ball or condyle) IVJs and horizontally oriented zygapophyses, allowing a more flexible arrangement of bony scutes (or osteoderms) along their backs and therefore greater trunk flexibility, particularly in the mediolateral direction. In contrast, extinct crocodylomorphs with amphicoelous (biconcave) IVJs relied to a greater extent on the interlocking double row of osteoderms that run along their back—known as the paravertebral shield—for support. This arrangement would have sharply limited mediolateral trunk flexibility, particularly in early crocodylomorphs, most of which had very rigid paravertebral shields. Thalattosuchians lacked both procoelous IVJs and interlocking osteoderms (although teleosaurid thalattosuchians had osteoderms, they did not interlock like those of most early crocodylomorphs), so the ability for sustained terrestrial locomotion would have been restricted to smaller animals [21].

To test whether vertebral morphology correlates with locomotor potential and associated ecological behaviours in extinct and extant crocodylomorphs, we measured osteological RoM and took morphometric measurements from three-dimensional virtual models of thoracic and lumbar vertebrae of four major groups of the Crocodylomorpha: ‘sphenosuchians’, ‘protosuchians’, Thalattosuchia and Crocodylia (table 1). *Terrestrisuchus* *gracilis* is a ‘sphenosuchian’-grade crocodylomorph, potentially near the origin of Crocodyliformes [22]; its locomotion is reconstructed as being upright, terrestrial and possibly digitigrade [8]. *Protosuchus* *richardsoni* represents a slightly more crownward ‘protosuchian’ crocodyliform with limb proportions and presumed limb postures and degrees of terrestrial locomotor ability intermediate between those of early crocodylomorphs and modern crocodylians [23]. We also included three species of thalattosuchians with increasing degrees of aquatic adaptation. *Pelagosaurus typus* is a small, semi-aquatic thalattosuchian that would have retained the ability to move on land [24], *Steneosaurus leedsi* is a large teleosaurid thalattosuchian that would have been confined to near shore environments, and *Metriorhychus superciliosus* is a medium-sized metriorhynchid thalattosuchian that is hypothesized to have been a pelagic predator [25–27]. Compared to *Pelagosaurus* and *Steneosaurus*, *Metriorynchus* is more specialized for aquatic locomotion with hydrofoil-like limbs, complete loss of osteoderms and a fishlike tail [28]. Finally, we included *Crocodylus niloticus* as a representative of modern semi-aquatic crocodylians, considering that this genus is also capable of bounding and galloping gaits at small body mass (in juveniles and smaller adults [29,30]).

**Table 1.** Crocodylomorph specimens used for morphometric and RoM analyses. Vertebral count is the total number of thoracic and lumbar vertebrae; figures in parentheses represent number of thoracic vertebrae (first) and lumbar (second) in taxa that have distinct thoracic and lumbar regions. Column length is summed centrum length (CL), and intervertebral (IV) space was measured from joints used to estimate RoM, excluding lumbosacral joint. Voxel size (v.s.) is the same in all dimensions. ‘Average number of mesh faces’ refers to the STL files exported from Mimics. IV space in *Steneosaurus* is not applicable (n.a.) because it is disarticulated and we did not model joints. Institutional abbreviations: American Museum of Natural History, New York (AMNH), Natural History Museum, London (NHMUK), Royal Veterinary College, London (RVC), Stony Brook University, New York (SBU).

taxon	type	source	scan		vertebral count	column length and range of CL (cm)	scan parameters	avg. no. mesh faces	IV space (cm) and as % of CL
*Terrestrisuchus gracilis*	fossil	NHMUK PV R 7562	μCT	NHMUK	(15^*a*^ (10^*a*^,5)	unknown (0.99–1.03)	190 kVp, 200 μA, v.s. 0.06 mm	61K	0.04 (4%)
*Protosuchus richardsoni*	fossil	AMNH 3024	CT	SBU	15 (10,5)	13.8 (0.88–1.31)	140 kVp, 300 mA, v.s. 0.39 mm	23K	0.12–0.17 (12.5%)
*Pelagosaurus typus*	fossil	NHMUK PV OR 32598	μCT	NHMUK	15 (10,5)	31.7 (1.73–2.29)	200–210 kVp, 200–220 μA, v.s. 0.08 mm	145K	0.07–0.12 (9%)
*Metriorhynchus superciliosus*	fossil	NHMUK R 2054	CT	RVC	17	66.3 (3.14–4.14)	100 kVp, 200 mA, v.s. 0.36 mm	76K	0.42–0.63 (13.4%)
*Steneosaurus leedsi*	fossil	NHMUK R 3806	CT	RVC	13	62.7 (5.02–5.47)	100 kVp, 200 mA, v.s. 0.32 mm	89K	n.a.
*Crocodylus niloticus*	frozen; recent	La Ferme aux Crocodiles	CT	RVC	15 (10,5)	52.7 (1.64–1.94)	120 kVp, 100 mA, v.s. 0.41 mm	57K	0.48–0.59 (19%)

^*a*^Thoracic vertebral number for *Terrestrisuchus* is reconstructed based on related taxa [8].

Based on our taxon sampling, we hypothesized that vertebral morphometric parameters known to correlate with mediolateral stiffness [20] would be more pronounced (and those correlated with dorsoventral stiffness would be less pronounced) in early crocodylomorphs (e.g. *Terrestrisuchus*, *Protosuchus*) as compared with thalattosuchians and modern crocodylians. This hypothesis is based on the idea that early crocodylomorphs were highly terrestrial and may have used asymmetrical gaits, a suite of behaviours requiring greater dorsoventral movements of the vertebral column than sprawling or swimming. Furthermore, we hypothesized that osteological RoM would be smaller in the mediolateral direction and greater in the dorsoventral direction in early crocodylomorphs, while the reverse would be evident in thalattosuchians and modern crocodylians. Finally, we hypothesized that pelagic thalattosuchians (i.e. metriorhynchids) would have morphometric parameters correlated with an increase in IVJ stiffness. The rationale for this hypothesis is that other pelagic marine reptiles, such as ichthyosaurs, sauropterygians and mosasaurs, are thought to have had relatively stiff vertebral columns (e.g. [31–34]), because such a morphology increases average swimming speed by increasing the natural frequency of body undulation at which the energetic cost of movement is minimized relative to speed [13,14,35].

## 2. Material and methods

### 2.1 Scanning, segmentation and retrodeformation

*Terrestrisuchus* and *Pelagosaurus* were micro-CT scanned at the Imaging and Analysis Centre in the Natural History Museum, London, UK (NHMUK) using a Metris X-Tek HMX ST 225 system. Scanning parameters for *Terrestrisuchus* were: 190 kVp, 200 μA, voxel size 0.06 mm in all dimensions. The three blocks containing *Pelagosaurus* were scanned separately, and the parameters ranged from: 200–210 kVp, 200–220 μA, voxel size 0.08 mm in all dimensions. The scans were reconstructed using CT PRO (Metris X-Tek, UK) and visualized using VG Studio Max v. 2.0 (Volume Graphics, Heidelberg, Germany). Dr Farah Ahmed and Mr Dan Sykes (NHMUK) and Dr Julia Molnar performed the scans, and Dr Molnar performed the reconstruction and visualization. *Protosuchus* was scanned by Dr Alan Turner at Stony Brook University Hospital using a LightSpeed VCT Scanner (GE Healthcare, Waukesha, WI, USA). Scan parameters were: 140 kVp, 300 mA, voxel size 0.39 mm in all dimensions. Multiple scans were taken to improve resolution. *Steneosaurus*, *Metriorhynchus* and *Crocodylus* were scanned by Dr John R. Hutchinson at the Royal Veterinary College using a medical CT scanner (GE Lightspeed) and reconstructed using Medview software (www.Medimage.com). Scanning parameters were, for *Steneosaurus*: 100 kVp, 200 mA, voxel size 0.32 mm in all dimensions; for *Metriorhynchus*: 100 kVp, 200 mA, voxel size 0.36 mm in all dimensions; and for *Crocodylus*: 120 kVp, 100 mA, voxel size 0.41 mm in all dimensions.

**Figure 1.**
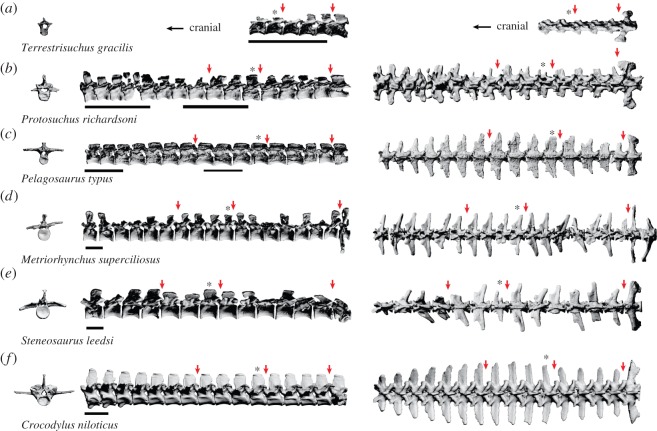
Virtual thoracolumbar vertebral columns of crocodylomorphs. From left: a single vertebra (*) in cranial view, the entire column in left lateral view, and the entire column in dorsal view. Arrows indicate the positions of joints (thoracic/cranial thoracic, lumbar/caudal thoracic, lumbosacral) for which RoM was estimated. Scale bars, 5 cm.

The reconstructed scans were segmented in Mimics (Materialise Inc. (www.materialise.com/mimics); Leeuwen, Belgium), a commercial image processing program for segmenting CT and MRI data and constructing triangulated three-dimensional surface meshes, to separate the fossil from the matrix. The fossils were segmented semi-automatically and each vertebra was volume-rendered, then its surface was exported as a separate STL file. Virtual bones were imported into 3D Studio Max (Autodesk 3ds Max; www.autodesk.com/3dsMax) and the joints were aligned to a standard coordinate system (figure 1).

Some degree of retrodeformation was required for *Metriorhynchus*, *Steneosaurus* and *Protosuchus.* The *Metriorhynchus* and *Steneosaurus* fossils were disarticulated, and each vertebra was deformed differently. Symmetry was restored using Landmark, a program with an algorithm designed for retrodeformation (Landmark; Institute for Data Analysis and Visualization, http://graphics.idav.ucdavis.edu/research/projects/EvoMorph). The *Protosuchus* specimen was articulated, but compressed dorsoventrally and mediolaterally. Retrodeformation using Landmark was attempted, but the software required each vertebra to be retrodeformed separately, resulting in mismatched articulations that would not fit back together. Instead, modelling tools in 3D Studio Max were used to scale the vertebrae in the direction of compression to restore symmetry and approximate the original dimensions (e.g. [36]). A sensitivity analysis showed that retrodeformation decreased RoM estimates by an average of less than 2° in each direction and that relative RoM in different directions was unaffected.

### 2.2 Morphometric measurements

To estimate relative joint stiffness, we compiled a list of 14 vertebral morphometric measurements linked to function through mechanical joint testing, correlation with locomotor behaviour, and/or engineering beam theory (table 2). Ten linear and four angular measurements were taken from all dorsal vertebrae. Morphometrics for *Crocodylus* were taken from Molnar *et al.* [20] and represent a single 10.1 kg juvenile specimen. For the remaining specimens, measurements were taken in ImageJ (http://rsbweb.nih.gov/ij) from orthographic projections of the virtual skeletons generated in 3D Studio Max. Linear measurements were normalized using the allometric scaling function:

$$M_{\mathrm{adj}}=M{\left(\frac{L_{s}}{L_{0}}\right)}^{b},$$

where *M*_adj_ is the normalized measurement, *M* is the original measurement, *L*_s_ is overall mean thoracolumbar length for all specimens (approximated by summing centrum length from each of the five lumbar vertebrae), *L*_0_ is lumbar length of the current specimen, and *b* is the slope of the regression of log_10_*M* on log_10_*L*_0_ for each measurement, using all specimens [19]. Using lumbar length rather than thoracolumbar length was a necessary simplification due to the incompleteness of the *Terrestrisuchus* specimen. Length of limb bones was not used for normalization because their lengths and proportions vary among the taxa analysed (e.g. ‘sphenosuchians’ have long slender limbs, while thalattosuchians have reduced limbs).

**Table 2.** Summary of relationships between vertebral morphology and stiffness/RoM from previous studies. Long *et al.* [14] and Molnar *et al.* [20] are experimental studies that measured stiffness/RoM. Measurements identified as useful predictors of passive IVJ stiffness and/or RoM in both mammals and crocodylians are shown in italics; see §3.1 for details. DE, dorsal extension, VF, ventral flexion, LF, lateral flexion; AR, axial rotation; CL, centrum length; TPW, transverse process width; CH, centrum height; CW, centrum width; LW, lamina width; NSH, neural spine height; A-NS, neural spine angle; NSL, neural spine length; A-TPD, dorsoventral transverse process angle; A-TPC, cranio-caudal transverse process angle; A-PZ, pre-zygapophyseal angle; PZW, pre-zygapophyseal width; IVD, intervertebral disc; IZL, inter-zygapophyseal length.

	morphological feature	correlation	direction	animal(s)	study
stiffness	*CH*	+	DE, VF	various mammals, Nile crocodiles	[15],[20],[37]^*a*^
	*CW*	+	LF	various mammals, Nile crocodiles	[15],[20],[37]^*a*^
	*LW*	+	DE, LF	various mammals, Nile crocodiles	[20]^*a*^,[37]^*a*^
	*A-PZ*	−	LF	Nile crocodiles	[20]
	*PZW*	+	DE, VF, LF	Nile crocodiles	[20]
	IVD length	−	DE	dolphins	[14]
	IVD width	+	DE	dolphins	[14]
	CL	−	DE, VF	dolphins	[14]
	TPW	+	LF	dolphins, seals	[14],[19]
	NSH	+	DE, VF	aquatic mammals	[14],[15]
	A-NS	−	VF, LF	Nile crocodiles	[20]
	A-TPD	+	DE, VF, LF	Nile crocodiles	[20]
RoM	*A-PZ*	+	LF	various mammals	[25,37^*a*^]
	*PZW*	−	AR	various mammals	[17]
	CL	−	DE, VF	whales, seals	[16,19]
	NSL	−	DE	various mammals	[38]
	A-TPD	+	DE, VF	various mammals	[37]^*a*^
	A-TPC	−	DE, VF	various mammals	[37]^*a*^
	A-NS	−	DE, VF	various mammals	[37]^*a*^
	IZL	+	DE, VF	Primates	[37^*a*^,^39^]

^*a*^And references therein.

### 2.3 Building virtual models

All fossil specimens used for RoM estimation, except *Metriorhynchus*, were preserved in articulation (both centra and zygapophyses), and the vertebral columns were fairly straight, suggesting that the intervertebral discs (IVDs) or other soft tissue between the vertebrae did not shrink extensively prior to fossilization. Therefore, the preserved space between adjoining centra probably represents a reasonable approximation of the thickness of IVDs (e.g. [40]). Vertebrae of *Metriorhynchus* were re-articulated by aligning the zygapophyseal facets with maximum overlap. Before manipulating the models to estimate RoM (see below), we defined a neutral pose and centre of rotation (CoR).

#### 2.3.1 Neutral pose

In their work on sauropod necks, Stevens & Parrish [41] defined neutral poses where the pre- and post-zygapophyses overlap maximally and the margin of the cotyle of the cranial vertebra is parallel to the capsule scar of the caudal vertebra in the coronal plane. For platycoelous centra, the facets of the centra would be parallel in this model. Based upon observations of ostriches, camels and giraffes, Christian & Dzemski [42] argued that the margins of the centra are not parallel when the zygapophyses are aligned and that they often are not parallel in habitual or resting poses. We do not consider the neutral pose in a behavioural context (e.g. ‘resting pose’ or habitual posture), but only as a starting point from which to measure joint deflection. Therefore, we used a variation of the definition of Stevens & Parrish [41] because it is easy to consistently reproduce and is thought to represent an intermediate position within the joint's RoM [43]. Defining a consistent neutral pose is important in this study for two reasons: first, it allows comparison of relative RoM in ventral flexion versus dorsal extension across joints and species, and second, the joint is positioned in a ‘neutral’ dorsoventral joint angle when estimating mediolateral RoM, potentially affecting its magnitude (i.e. coupled motions).

For articulated specimens, the neutral pose was kept as similar to the preserved arrangement as possible. First, the vertebrae were linked hierarchically so that movement of one vertebra caused all vertebrae caudal to it to move along with it. Second, the cranialmost vertebra was rotated and translated to fit the following criteria:

(1) translate along the *X*- and *Z*-axes until the neural canal is centred on (0, 0) in the *X*–*Z* (coronal) plane;
(2) rotate around the *X*- and *Z*-axes until the long axis of the centrum is aligned with the global *Y* -axis (cranio-caudal axis); and
(3) rotate around the *Y* -axis until the left and right zygapophyses and transverse processes appear as mirror images in the *X*–*Z* (coronal) plane.

The next (caudally) vertebra was aligned following the same procedure after it had been linked to the CoR object (next section). Finally, that next vertebra was translated minimally along the *Y* -axis only if the two vertebrae were intersecting or disarticulated. The same procedure was followed for disarticulated specimens, except that translation along the *Y* -axis was performed until maximal zygapophyseal overlap was reached. This procedure was validated using three joints from a CT scan of a Nile crocodile cadaver: the virtual bones were duplicated and one pair was aligned visually. The average difference between the two treatments was very small: approximately 1.6% of centrum length (0.49 mm).

#### 2.3.2 Location of centre of rotation

We placed the CoR at the centre of the condyle (cranially) for procoelous vertebrae [44] or where the centre of the IVD would have been located [45]. A sphere was fitted to the centre of the condyle of the preceding vertebra (procoelous) or an ellipsoid was fitted to the space between the two centra (amphicoelous). A more ventral CoR has been reported in some human studies (e.g. [46]), so we performed a sensitivity analysis to assess how variations in the CoR location, as well as the amount of translation allowed, affected our results. The sensitivity analysis showed that this variable produced only minor effects which did not change any of our conclusions (table 3).

**Table 3.** Sensitivity analysis: differences in mean RoM for joint L1–2 by CoR location and translation. Mean RoM in degrees estimated from virtual models. For CoR location condyle: CoR is located at the condyle of the cranial vertebra for procoelous vertebrae or the centre of the IVD, where present. For CoR location NC: CoR is located at the anterior (ventral) margin of the spinal (neural) canal. NC, neural canal; ML, mediolateral; DE, dorsal extension; VF, ventral flexion; AR, axial rotation; 50% Z.O., minimum 50% zygapophyseal overlap.

	translation allowed			CoR location		
	0%	1.50%	3%	condyle	NC	50% Z.O.
	*Terrestrisuchus*					
ML	6.23	11.18	17.71	11.71	11.71	8.6
DE	0.42	3.79	7.69	3.79	4.13	3.79
VF	2.12	6.14	9.26	5.67	6.02	3.25
AR	0.34	1.41	2.51	1.26	1.59	1.26
average	2.28	5.63	9.29	5.61	5.86	4.23
	*Protosuchus*					
ML	8.45	16.02	21.45	15.31	15.31	13.08
DE	2.44	5.55	8.59	5.19	5.87	5.19
VF	4.77	9.94	11.87	9.08	8.64	2.22
AR	1.23	2.2	3.1	2.06	2.29	2.06
average	4.22	8.43	11.25	7.91	8.03	5.64
	*Pelagosaurus*					
ML	10.68	15.06	19.31	15.02	15.02	12.9
DE	4.17	5.46	7.21	4.2	7.03	4.2
VF	10.68	12.36	13.21	15.37	8.8	1.64
AR	1.71	2.54	3.34	2.19	2.86	2.19
average	6.81	8.85	10.77	9.19	8.43	5.23
	*Metriorhynchus*					
ML	14.53	18.78	8.17			16.33
DE	1.12	4.12	4.12			1.12
VF	10.54	11.34	3.97			3.97
AR	3.64	7.44	7.44			7.44
average	7.46	10.42	5.93			7.22
	*Crocodylus*					
ML	16.98	20.19	21.53	19.56	19.56	7.92
DE	11.69	12.7	13.75	9.43	16	9.43
VF	7.49	11.45	13.35	17.47	4.05	2.47
AR	2.45	2.87	3.24	2.63	3.08	2.63
average	9.65	11.8	12.97	12.27	10.67	5.61

#### 2.3.3 Estimating osteological range of motion

RoM in ventral flexion, dorsal extension and lateral flexion was estimated by manipulating the virtual fossils in 3d Studio Max. The preceding vertebra was held stationary, while the vertebra caudal to it was rotated about its CoR until it reached an osteological stop (i.e. the bones intersected) or the joint disarticulated (i.e. zygapophyseal facets no longer overlapped). Contacts between bones were detected visually, checking that the vertebrae did not touch or penetrate each other in three dimensions. We examined the effect of setting zygapophyseal overlap minima of both 0% (no overlap) and 50% to cover the range found in previous studies [41,42,47] (table 3). Additionally, we did not allow separation of zygapophyseal facets in the dorsoventral direction because disarticulation of zygapophyses would cause instability and would be unlikely to occur during normal locomotion. A small amount of translation between vertebrae (1.5% centrum length) and up to 3° of rotation about the long axis of the column (torsion) were allowed. Three degrees of torsion was chosen as a conservative value to allow the maximum possible flexion/extension; torsion during walking in extant alligators averages only about 10° across the entire trunk [48]. Some translation almost certainly occurs at IVJs, because the soft tissues of the joint deform under pressure. In human lumbar IVJs, an average of 0.3–2.0 mm translation was measured in concert with mediolateral and dorsoventral bending [49,50]. For comparison, 1 mm is about 3.3% of centrum length in the human lumbar vertebrae used by Xia *et al.* [50]. A sensitivity analysis was conducted to assess the effects of amounts of translation on RoM estimates (table 3). We were not able to include osteoderms in this study because only two of the fossil specimens had osteoderms preserved.

### 2.4 Sequential removal of axial tissues in *Crocodylus*

**Figure 2.**
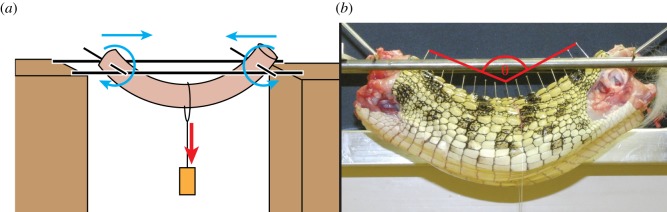
Experimental set-up for whole trunk bending in *Crocodylus* (*a*) and photograph of whole trunk in dorsal extension (*b*). Red arrow shows the application of force, and blue arrows show the direction of movement of the two ends of the specimen; *θ* is trunk angle measured from position of pins.

Deflection and stiffness of the whole trunk were measured, both with and without soft tissues, for two juvenile *C. niloticus* specimens with body masses of 1.6 and 2.2 kg. The specimens were provided by the conservation centre La Ferme aux Crocodiles (Pierrelatte, France), had died of natural causes and were sealed in airtight plastic bags and frozen (−20°C) immediately thereafter. They were thawed at room temperature for approximately 24 h before dissection. The tail, head and limbs were removed, leaving the last cervical and first caudal vertebrae intact. These two vertebrae were stripped of flesh and holes were drilled through them in the mediolateral and dorsoventral directions to accommodate rods used for suspension. Sewing pins were inserted between the osteoderms and into the neural spines of each vertebra to track its movement. The tenth thoracic (i.e. dorsal) vertebra was located by counting rows of osteoderms, and its position was marked on the skin to approximate the midpoint of the trunk. Each specimen was weighed. Thin metal rods were passed through the drilled holes on either end of the specimen, and the rods were balanced on two larger rods running parallel to the length of the specimen (figure 2). The diameter of the rods was smaller than the drilled holes, so the vertebrae were able to rotate freely. Also, the thinner rods were able to slide along the length of the thicker rods, meaning that the apparatus did not impede flexion of the specimen or rotation about either end. A length of fishing line was looped around the midpoint of the trunk, and metric weights were suspended from the fishing line, causing the trunk to flex and its ends to move closer to each other.

The specimen was loaded and deflections were recorded as described in Molnar *et al.* [20]. Ten different weights were used: 0.02, 0.05, 0.1, 0.2, 0.3, 0.4, 0.5, 0.6, 0.7 and 0.8 kg. The entire procedure was performed three times for each specimen in ventral flexion, dorsal extension and lateral flexion. Soft tissues were removed sequentially, and measurements were repeated after each step, as follows:

(1) whole trunk;
(2) skin removed (osteoderms left intact);
(3) viscera removed;
(4) ribs removed;
(5) accessory osteoderms removed;
(6) paravertebral osteoderms (two sagittal rows either side of the midline) removed; and
(7) epaxial muscles removed.

Deflection angles were measured between the cranialmost, caudalmost and central pins, the central pin having been placed in the neural spine of the tenth thoracic vertebra (figure 2*b*). Measurements were taken from photographs (to scale) in ImageJ software.

The applied moment (*M*) was then calculated using the following equation:

$$M=(m_{w}+m_{s})\cdot g\cdot \left(\frac{L}{2\sin(\theta /2)}\right),$$

where the applied force is the sum of the mass of the added weight (*m*_*w*_) and the specimen mass in kilograms (*m*_s_) times *g* (=9.81 ms^−2^), and the moment arm of the applied force was calculated from specimen length in metres (*L*) and trunk angle in degrees (*θ*) using trigonometry. To simplify calculations, it was assumed that specimen length does not change and that forces act on the specimen at the base of the central pin.

Stiffness was calculated as described in Molnar *et al.* [20]. Briefly, the rotational force applied to the specimen in Newton metres (moment; *M*) was plotted against trunk angle in degrees, where 180° represents a flat trunk with no deflection in any direction. Because the mass of the specimen changed substantially with the removal of tissues, it was not possible to choose a range of equivalent moments across different treatments. Therefore, measured points from the approximately linear region of the moment–deflection graph with moments above 0.3 Nm were used to fit the regression lines that defined stiffness. Differences in deflection produced by equivalent moments (‘trunk deflection’) are represented by the *x*-intercept of the regression line, but they are not equivalent to neutral zones because very small moments were not measured in most cases.

## 3. Results

### 3.1 Estimates of thoracolumbar intervertebral joint stiffness based on morphometrics

**Figure 3.**
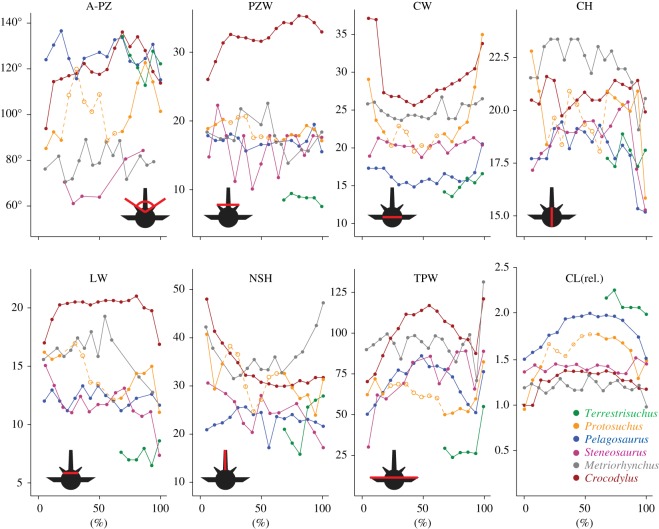
Normalized morphometric measurements from thoracic and lumbar vertebrae. Length units not shown because measurements were normalized; see electronic supplementary material, table S2. The *x*-axis shows percentage of trunk length: 0% is the first thoracic, 100% is the first sacral. Dashed lines represent regions of poor preservation. Diagrams show vertebra from cranial view, and red lines show how each measurement was taken. A-PZ, pre-zygapophyseal angle (not normalized); PZW, pre-zygapophyseal width, i.e. mediolateral spacing of pre-zygapophyses; CW, centrum width; CH, centrum height; LW, lamina width; NSH, neural spine height; TPW, transverse process width; CL(rel.) relative centrum length = 2×length/(centrum width+height).

Within each of the three crocodylomorph groups examined (early crocodylomorphs, thalattosuchians and modern crocodylian), the vertebrae share many morphological characteristics. The early crocodylomorph vertebrae are characterized by relatively long centra, short vertebral processes (neural spines and transverse processes) and medium-sized zygapophyseal joints, qualitatively similar to the ancestral archosaurian state (JR Molnar & JR Hutchinson 2015, personal observation). The thalattosuchian vertebrae have shorter centra (except *Pelagosaurus*), long, slender vertebral processes, and small zygapophyses. The modern crocodylian vertebrae have short, procoelous centra that are wider than they are tall, long, robust vertebral processes, and large, widely spaced zygapophyses (figures 1 and 3).

Based on previous studies, five morphometric measurements appear to be the most useful predictors of passive IVJ stiffness and/or RoM in both mammals and crocodylians (italic font in table 2). Correlates of high mediolateral stiffness include vertically oriented zygapophyses (smaller pre-zygapophyseal angles), widely spaced zygapophyses and mediolaterally wide centra and laminae. Correlates of high dorsoventral stiffness include horizontally oriented zygapophyses (larger pre-zygapophyseal angles) and dorsoventrally tall centra [20]. In addition, the length of neural spines and transverse processes give an idea of the cross-sectional areas and leverages of axial muscles, which would increase passive stiffness in the intact animal [19].

The morphometrics of the lumbar region in *Terrestrisuchus* (figure 3) are consistent with low stiffness, particularly in the mediolateral direction. Its narrowly spaced zygapophyses and narrow laminae imply low mediolateral stiffness, and its pre-zygapophyseal angles well over 90° suggest greater dorsoventral than mediolateral stiffness. *Protosuchus* is intermediate in almost every measurement, although the taphonomic distortion of the fossil and the low resolution of the scans make the morphometrics somewhat difficult to interpret. Its transverse processes are relatively short, suggesting that it lacked the muscle mass and leverage for powerful lateral flexion. The relative centrum dimensions of *Protosuchus* were most similar to *Steneosaurus*, but the zygapophyses of the former were closer to horizontal (suggesting greater dorsoventral stiffness) and its transverse processes did not become broader in the middle of the column. *Pelagosaurus* seems to have low mediolateral and intermediate dorsoventral stiffness: its zygapophyses are sub-horizontal, suggesting greater dorsoventral than mediolateral stiffness, and the centra are spool-shaped with a small diameter relative to their length, suggesting low overall stiffness. In contrast, *Steneosaurus* has more vertical zygapophyses and shorter, slightly wider centra, suggesting greater mediolateral than dorsoventral stiffness; other morphometrics are similar to *Pelagosaurus*. *Metriorhynchus* also has more vertical zygapophyses, but it has much taller, wider centra suggesting higher overall stiffness. *Crocodylus* has widely spaced, sub-horizontal zygapophyses, broad laminae and very short, wide centra, suggesting high mediolateral and dorsoventral stiffness.

### 3.2 Osteological range of motion estimated from virtual skeletons

**Figure 4.**
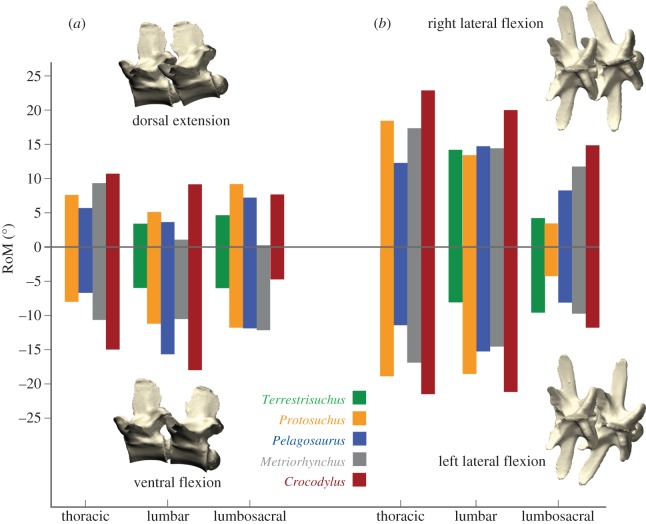
Maximum osteological RoM estimated by manipulation of virtual models. Estimates shown in (*a*) dorsoventral and (*b*) mediolateral flexion for three dorsal IVJs (except *Terrestrisuchus*, which lacked a thoracic region). *Steneosaurus* was not included because the zygapophyses were deformed and could not be articulated. *Crocodylus* vertebrae are shown.

Despite their morphological differences, estimated RoM was surprisingly similar across the five taxa. No specimen stood out as being remarkably different from the others in overall RoM, osteological limitations to RoM, or the effects of variation in CoR and allowed translation amounts (tables 3 and 4). However, some taxa clearly had greater RoM than others in certain directions, and in many cases RoM was substantially different between the lumbosacral joint and the other two joints chosen to represent different regions of the trunk (figure 4). *Crocodylus*had the greatest RoM on average in both planes of movement for all three joints tested (figure 4), but the most restricted RoM in ventral flexion at the lumbosacral joint. Based on the caudalmost two joints, *Terrestrisuchus* had the smallest average RoM in every direction except dorsal extension, where *Metriorhynchus* had the smallest RoM. In most cases, RoM was greater in lateral flexion than dorsal or ventral flexion. However, the average RoM in ventral flexion for *Pelagosaurus* was similar to average lateral flexion RoM (11.4° versus 11.7°), and *Metriorhynchus*, *Pelagosaurus* and *Protosuchus* had greater RoM in ventral than lateral flexion at the lumbosacral joint. RoM in lateral flexion was generally fairly symmetrical, except in *Terrestrisuchus* where asymmetrical preservation caused the joints to reach osteological stops at different degrees of flexion to the left and right. The lumbosacral joints of all taxa had notably lower lateral RoM than the lumbar joints (3.9–13.4° versus 11.2–20.2°). RoM was similar between the thoracic and lumbar joints except in *Metriorhynchus*, where dorsal extension was greater in the thoracic joint. See electronic supplementary material, table S2, for non-normalized measurements.

The amount of space between adjacent centra relative to centrum length was substantially greater in *Crocodylus* than the other taxa, and substantially smaller in *Terrestrisuchus* (table 1). This difference might be related to preservation (in the case of *Terrestrisuchus*), or it might reflect different amounts of soft tissue within the joints (more likely in the case of *Crocodylus* because it is the only studied taxon with procoelous IVJs). If it is an artefact of preservation, the small amount of intervertebral space in *Terrestrisuchus* might cause our RoM estimates to be too low, particularly in lateral flexion because this movement was limited by contact between the centra (table 4).

**Table 4.** Osteological RoM and structures that limit RoM. Summary of estimated RoM values. Values for ML and AR are averages of left and right RoM. Mean RoM in degrees and limitations on RoM (in italics) in each taxon, joint and direction. ZI, zygapophyses intersect; ZDl, zygapophyses disarticulate in the mediolateral, dorsoventral (ZDd) or cranio-caudal (ZDc) direction; CI, centra intersect.

	lateral flexion		dorsal extension		ventral flexion	
	*Terrestrisuchus*					
lumbar	11.2°	*CI*	3.5°	*ZI*	6.0°	*ZD*(*d*)
lumbosacral	6.9°	*CI*	4.7°	*ZI*	6.0°	*ZD*(*d*), *CI*
	*Protosuchus*					
thoracic	18.7°	*CI*, *ZD*(*l*)	7.7°	*ZI*	8.0°	*ZD*(*d*)
lumbar	16.0°	*CI*, *ZD*(*l*)	5.2°	*ZI*	11.2°	*ZD*(*d*)
lumbosacral	3.9°	*CI*	9.3°	*ZI*	11.8°	*ZD*(*d*), *CI*
	*Pelagosaurus*					
cranial thoracic	11.9°	*CI*	5.8°	*ZI*, *CI*	6.7°	*CI*
caudal thoracic	15.1°	*ZI*, *ZD*(*d*)	3.7°	*ZI*	15.7°	*CI*, *ZD*(*c*)
lumbosacral	8.2°	*CI*	7.3°	*ZI*, *CI*	11.9°	*CI*
	*Metriorhynchus*					
cranial thoracic	17.2°	*CI*	9.4°	*ZI*	10.7°	*ZD*(*d*,*l*)
caudal thoracic	14.5°	*ZI*, *CI*	1.1°	*ZI*	10.5°	*ZD*(*d*,*l*)
lumbosacral	10.8°	*ZI*, *CI*	0.3°	*ZI*	12.2°	*ZI*, *CI*
	*Crocodylus*					
thoracic	22.3°	*ZD*(*l*), *ZI*	11.0°	*ZI*	15.1°	*ZD*(*d*)
lumbar	20.2°	*ZD*(*l*), *ZI*	9.4°	*CI*	18.2°	*ZD*(*d*, *l*), *CI*
lumbosacral	13.4°	*ZD*(*l*), *ZI*	7.9°	*ZI*, *CI*	4.7°	*ZD*(*d*)
average	18.6°		9.4°		12.7°	

**Figure 5.**
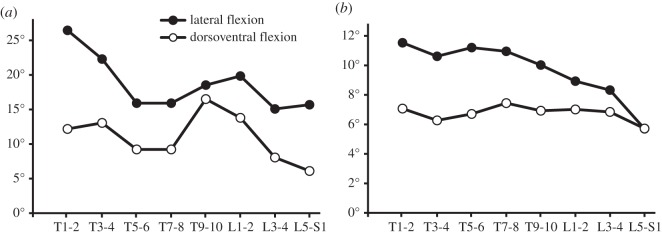
Validation of RoM estimation method by qualitative comparison with experimental data. Patterns of RoM along the thoracolumbar vertebral column in *Crocodylus* in dorsoventral and mediolateral flexion estimated from manipulation of virtual skeletons of *Crocodylus* allowing 0.45 mm translation (*a*) and experimental measurements from individual cadaveric joints taken from Molnar *et al.* [20] (*b*). The *x*-axis shows joints (e.g. T1–2 is the joint between the first and second thoracic vertebrae), and the *y*-axis shows flexion in degrees. CoR was located at the condyle of the cranial vertebra and minimum zygapophyseal facet overlap was 1%. Both sets of results showed greater average RoM in lateral flexion than dorsoventral flexion; RoM in both directions decreased along the vertebral column—lateral flexion more steeply—whereas RoM in dorsoventral flexion peaked in the mid-trunk. The data from the virtual model were noisier and did not capture the more subtle differences, which was expected considering that only one specimen was scanned for the virtual model, whereas the experimental data represent averages from seven specimens.

Comparison of our virtual RoM *Crocodylus* data with those data recovered experimentally in cadaveric specimens showed that the virtual RoM estimation method successfully captured much of the variation between bending directions and along the vertebral column (figure 5).

### 3.3 Effects of other axial tissues in *Crocodylus*

**Figure 6.**
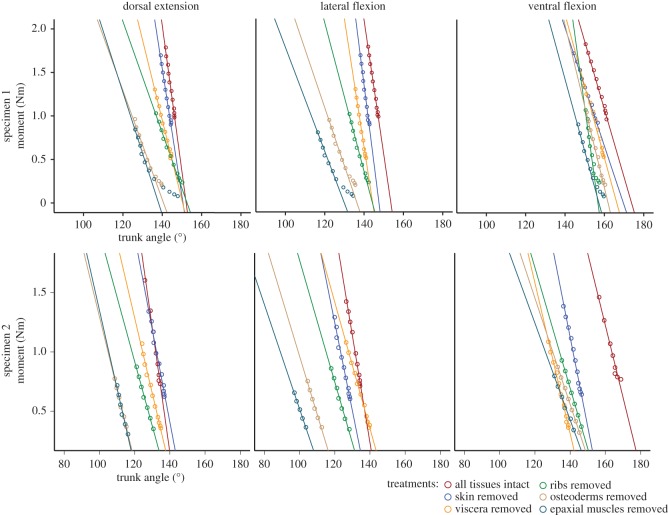
Moments versus trunk deflection for whole trunks with sequential removal of tissues in *Crocodylus*. Moments in Newton metres, angles in degrees. Trunk angle of 180° represents zero deflection. Lines are linear regressions on all points for which moment is greater than 0.3 Nm. Combining data for the two specimens masked patterns of variation, so results are presented separately.

Removal of osteoderms, ribs and soft tissues substantially increased RoM and decreased stiffness of the trunk, and these effects varied across different bending directions. Removal of tissues had the greatest effect upon trunk deflection in lateral flexion, increasing it by a total of 12.3° versus 8.3° in ventral flexion and 9.8° in dorsal extension. Removal of tissues decreased stiffness by 58–61% in dorsal extension and lateral flexion, but only 6% in ventral flexion (table 5 and figure 6).

**Table 5.** Change in stiffness with sequential removal of axial tissues in *Crocodylus.* Coefficients (stiffness) and *x*-intercepts of linear regressions with trunk deflection (*Crocodylus*) in degrees as the independent variable and applied moment in Newton metres as the dependent variable. Only moments greater than 0.3 Nm were used in the regression.

		dorsal extension		lateral flexion		ventral flexion	
treatment	specimen mass (kg)	stiffness	*x*-intercept	stiffness	*x*-intercept	stiffness	*x*-intercept
whole trunk	0.95	0.168	29.7	0.154	35.8	0.078	13.6
skin removed	0.875	0.146	26.8	0.179	35.4	0.068	21.9
viscera removed	0.50	0.092	31.2	0.147	41.9	0.082	14.8
ribs removed	0.225	0.064	37.5	0.085	41.7	0.165	17.7
accessory osteoderms removed	0.20	0.083	33.6	0.076	45.8	0.139	20.2
paravertebral osteoderms removed	0.20	0.063	30.9	0.067	37.1	0.094	14.9
epaxial muscles removed	0.075	0.070	180	0.060	180	0.083	180

## 4. Discussion

**Figure 7.**
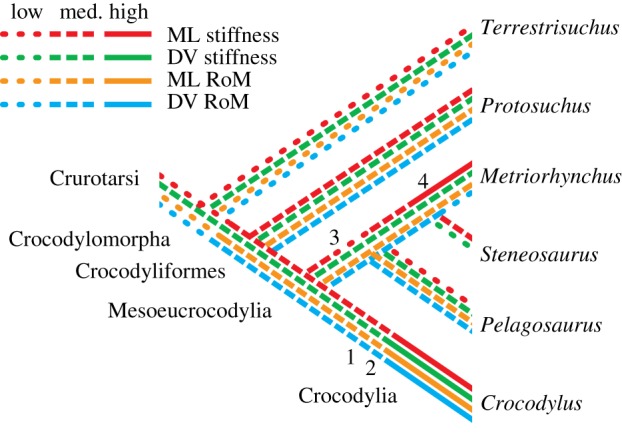
Hypothetical changes in IVJ stiffness and RoM with aquatic adaptation in Crocodylomorpha (phylogeny from [52]). Numbers show changes in other axial tissues: (1) sagittal segmentation of the paravertebral shield; (2) procoelous vertebrae; (3) ‘open’ margins of the paravertebral shield; (4) complete loss of osteoderms [21]. NB: procoelous vertebrae and/or loss of osteoderms evolved convergently in *Junggarsuchus* [7] and a few other crocodylomorph clades as well ([53] and references therein). Some of these hypotheses are sensitive to the phylogenetic placement of Thalattosuchia; see the electronic supplementary material for an alternative phylogeny that places Thalattosuchia outside Crocodyliformes. ML, mediolateral; DV, dorsoventral.

The crocodylomorph lineage includes species with locomotor habits ranging from fully aquatic (e.g. *Metriorhynchus*) to highly terrestrial (e.g. *Terrestrisuchus*). Sprawling and semi-aquatic locomotion have been associated with mediolateral movements of the vertebral column in crocodile-line archosaurs [51], whereas asymmetrical gaits seem be associated with dorsoventral movements [21]. For these reasons, we hypothesized that *Terrestrisuchus* and *Protosuchus* would have lower IVJ stiffness and greater RoM in the dorsoventral direction, and *Pelagosaurus*, *Steneosaurus* and *Metriorhynchus* would have lower stiffness and greater RoM in the mediolateral direction (*Metriorhynchus*was hypothesized to have higher overall stiffness because it is reconstructed as a pelagic pursuit predator). While the results for the thalattosuchians and *Crocodylus* generally matched our predictions, those for the early crocodylomorphs did not. The most likely explanation for this discrepancy is the effect of other axial structures such as skin, muscles and particularly the paravertebral shields of most early crocodylomorphs on IVJ movements, which are best examined here from an evolutionary perspective (figure 7).

### 4.1 Evolution of vertebral functional morphology in crocodylomorphs

#### 4.1.1 Early crocodylomorphs

We predicted that the early crocodylomorphs would have lower stiffness and greater RoM in the dorsoventral direction because members of this group are widely thought to have used parasagittal gaits based on the shapes and proportions of their limb bones and joints (e.g. [1,7–10]). In addition, analysis of pace angulation of trackways supports the inference that erect limb posture was employed by crocodile-line archosaurs in the Early Triassic [11], and early crocodylomorphs are primarily found in association with terrestrial rather than aquatic faunae [1]. Frey [54] postulated that the morphology of the trunk of *Protosuchus* was specialized for fast terrestrial locomotion and would not have allowed substantial lateral undulation (or adept swimming), in contrast to the morphology of modern crocodylians, which mainly permits mediolateral movements during terrestrial and aquatic locomotion. However, our estimates of IVJ stiffness and RoM do not reflect this pattern. On the contrary, average mediolateral RoM in the early crocodylomorphs was greater than dorsoventral RoM (similar to the other taxa in this study), and dorsoventral stiffness was estimated to be higher than mediolateral stiffness. These patterns were more pronounced in *Terrestrisuchus*: RoM in ventral flexion was sharply limited (6° maximum) by separation of the zygapophyseal facets; dorsoventral stiffness would have been increased by the near-horizontal zygapophyses; and mediolateral stiffness would have been reduced by the narrow spacing of the zygapophyses and narrow laminae and centra. The paravertebral shield would have further restricted movements and increased stiffness (see ‘Effects of other axial tissues’ below). While we only studied two early crocodylomorphs, the similar features of *Terrestrisuchus* and *Protosuchus* provide strong qualitative support that relatively greater IVJ stiffness and smaller RoM were ancestral for the crocodylomorph lineage (figure 7).

#### 4.1.2 Thalattosuchians

Thalattosuchians were characterized by short, flat limbs; furthermore, metriorhynchid thalattosuchians, reconstructed as specialized aquatic pursuit predators [55–57], had aquatic adaptations including paddle-like limbs, a streamlined body shape, a hypocercal tail [24,28,58] and osteoporotic lightening of the skeleton [59]. In agreement with our predictions, the thalattosuchians we examined had greater mediolateral than dorsoventral osteological RoM (figure 4) and estimated stiffness was greater in the dorsoventral than mediolateral direction in *Pelagosaurus* and greater overall in *Metriorhynchus* than the other taxa. The results for *Pelagosaurus* fit our predictions well: mediolateral stiffness was estimated to be lower than dorsoventral stiffness and RoM was greater in mediolateral flexion, similar to the semi-aquatic *Crocodylus*. On the contrary, the more vertically oriented zygapophyses and relatively low centra of *Steneosaurus* compared to the other taxa, including *Crocodylus* (figure 3), suggest greater stiffness in the mediolateral direction as compared to the dorsoventral direction. Since *Steneosaurus* probably spent most of its time in near shore environments, stiffness of the trunk in the mediolateral direction may have stabilized the body to allow fast lateral sweeping movements of the neck during prey capture. As noted by Hua [25], the cervical vertebrae of *Steneosaurus* had more horizontal zygapophyses, suggesting greater mediolateral RoM and lower stiffness. *Metriorhynchus* had smaller than average RoM in the dorsoventral direction, particularly in dorsal extension, which was limited by the zygapophyses (although more vertical than horizontal, their facets could not slide far in this direction before contacting the neural spine of the adjacent vertebra). Similar to *Steneosaurus*, estimated stiffness in *Metriorhynchus* was greater in the mediolateral than dorsoventral direction, due primarily to the more vertical orientation of their zygapophyses. This characteristic was also noted by Hua [25], who interpreted it to mean that *Steneosaurus*and *Metriorhynchus* had stiffer bodies than modern crocodylians. While our morphometric data generally agree that *Metriorhynchus* would have had relatively stiff IVJs, in our virtual models it was the large, flat central articulations rather than the zygapophyses that constituted the major restriction on lateral flexion. The zygapophyses of *Metriorhynchus* are small and close to the midline, meaning that large degrees of IVJ flexion require only small displacements of the zygapophyseal facets. Likewise, IVJ stiffness in *Metriorhynchus* would be conferred by resistance to compression of the soft tissue between the centra and by the large axial muscles implied by its long vertebral processes, in addition to stretching of the zygapophyseal joint capsules. We were not able to estimate RoM for *Steneosaurus*, but the preserved morphology qualitatively indicates a RoM within the range observed for the other two thalattosuchians.

#### 4.1.3 Modern crocodylians

In *Crocodylus*, our estimates from virtual models and morphometrics agree with experimental tests [20] showing that stiffness is lower and RoM is greater in the mediolateral direction (figure 4). This result is consistent with our expectations based on studies (cited below) showing that extant crocodylians use more mediolateral than dorsoventral flexion during locomotion. Lateral trunk flexion of 20–30° (left plus right) has been observed during terrestrial locomotion [48,60,61], and up to 90° (unilaterally) during swimming [21]. Dorsoventral flexion of about 5° (dorsal plus ventral) was recorded during a high walk [48], and some (presumably greater) dorsoventral flexion has been observed during galloping/bounding ([21] and references therein; [29]).

### 4.2 Implications for locomotion

Cross-sectional areas and leverages of axial muscles in crocodylomorphs, approximated by the lengths and orientations of vertebral processes, were largely consistent with presumed locomotor strategies. In more erect limb postures, such as those widely reconstructed for early crocodylomorphs, a greater component of ground reaction force acts on the vertebral column in the sagittal direction, whereas in more sprawling postures, such as those reconstructed for the remaining taxa, mediolateral components are greater (Crocodylia actually use a variety of ‘semi-erect’ postures [60]). In (more upright) mammals, axial muscles that stiffen the trunk in the dorsoventral direction are larger than in other tetrapods, and these muscles are bilaterally active during symmetrical gaits [62]. As expected, the aquatic and semi-aquatic crocodylomorph taxa had relatively broad transverse processes (figure 3), increasing the leverage of lateral flexors, and *Protosuchus* had relatively tall neural spines, increasing the leverage of dorsal extensors. *Terrestrisuchus* did not have particularly tall neural spines, but its transverse processes were even shorter. The more powerful active axial movements implied by the long vertebral processes of *Crocodylus* and *Metriorhynchus* may reflect the relative importance of axial movements, which decreases over the continuum of sprawling to erect locomotion [63] and might play a major role in undulation and/or stiffening of the trunk during swimming. The shorter vertebral processes of *Terrestrisuchus* and *Protosuchus* suggest that they relied to a greater extent on passive stabilization mechanisms such as the paravertebral shield. Alternatively or in combination, limb propulsion as opposed to trunk undulation may have contributed a greater proportion of locomotor power in these taxa; early crocodylomorphs, particularly the earlier non-crocodyliform lineages, had relatively longer (cursorial), more robust limbs than thalattosuchians and extant crocodylians [1,7].

Body size is another factor that influences the effect of axial stiffness on locomotion. Because of the scaling relationship between cross-sectional area, which determines muscle and bone strength, and volume, which determines body mass, larger animals must compensate behaviourally or structurally to avoid dangerously high stresses during locomotion [64]. In *Alligator mississippiensis*, the lengths of the neural spines and transverse processes increase significantly relative to body length throughout ontogeny, which may be explained by an increase in axial muscle mass [65] and thus may help compensate for size-related changes in stress on the axial column. The early crocodylomorphs were much smaller than the thalattosuchians and most extant, adult crocodylians: *Terrestrisuchus* was only 49–77 cm long (but may not have been fully grown), and *Protosuchus* was closer to 80 cm long [8,23], whereas the other species reached 3 m or more. Therefore, smaller body mass may have compensated somewhat for the low stiffness and relatively short vertebral processes of *Terrestrisuchus* and *Protosuchus*, lending them greater agility than larger animals with similar anatomy. Similarly, small mammals tend to have more flexible vertebral columns than larger ones [66]. Due to these constraints, sustained terrestrial locomotion probably was not possible for larger crocodylomorphs that lacked both rigid osteoderm articulations and procoelous IVJ articulations, such as thalattosuchians and dyrosaurids [21,67].

Apart from body size-related constraints on locomotor abilities, there is little evidence that the biomechanics of the vertebral column changes over ontogeny in crocodylians. Smaller adults, including the West African dwarf crocodile (*Osteolaemus* *tetraspis*), have been observed bounding and galloping ([3] and references therein), and measurements of limb bones [68] suggest no major departure from isometry across ontogeny. Molnar *et al.* [20] did not report allometry in the vertebrae of *C. niloticus* over the size range they studied (1.4–15.6 kg). Ikejiri [65], who examined a larger number of individuals over a greater range of sizes, did find statistically significant vertebral allometry in *A. mississippiensis*, but not in any of the dimensions correlated with stiffness in *C. niloticus* [20] which were used to estimate stiffness in this study. Given that the fossil individuals we studied appear to have been adults or sub-adults, the characters we measured probably would not have changed substantially if the animals had lived longer. Moreover, in smaller bodied taxa such as *Terrestrisuchus* and perhaps *Protosuchus*, similar to the sizes of many early Crocodylomorpha in general, this issue of large body size-related ontogenetic declines in locomotor abilities is less of a concern.

**Figure 8.**
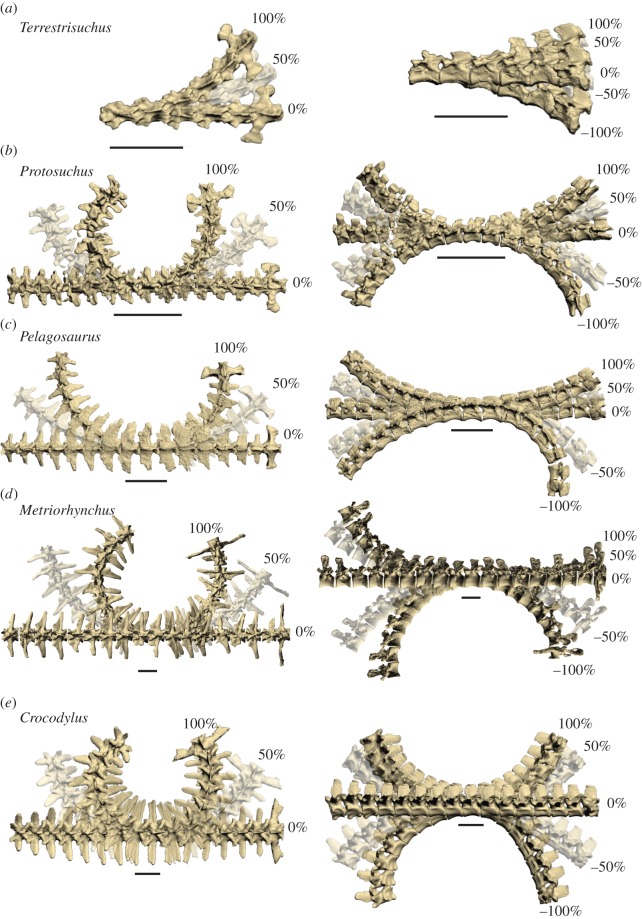
Osteological RoM estimates extrapolated to the entire thoracolumbar vertebral column. In the left column are dorsal views showing lateral flexion, and in the right column are lateral views showing dorsoventral flexion. Cranial is to the left. The RoM of each joint is equal to that of the nearest joint that we tested, disregarding the lumbosacral joint (RoM of the lumbosacral joint was applied only to that joint). Percentages are relative to maximum osteological RoM. Scale bar, 5 cm.

Finally, the total number of thoracolumbar vertebrae affects the behaviour of the trunk during locomotion. If RoM of individual joints remains constant, a greater number of dorsal vertebrae translates into greater RoM along the trunk [16]. Relative to *Protosuchus*, *Pelagosaurus* and *Crocodylus*, *Metriorhynchus* has two more thoracolumbar vertebrae and *Steneosaurus* has two fewer (table 1), presumably granting slightly greater total trunk RoM to the former and slightly less to the latter. Total trunk RoM for each taxon, extrapolated from the virtual joints we tested, is shown in figure 8.

#### 4.2.1 Bounding and galloping

The point at which asymmetrical gaits first evolved in the crocodylomorph lineage is not known [4]. Similarities in dorsoventral stiffness and RoM between *Terrestrisuchus*, *Protosuchus* and *Crocodylus* not shared by the thalattosuchians would support the idea that bounding and galloping in modern crocodylians is an ancestral trait inherited from their Triassic forebears and lost in more aquatic thalattosuchians. The lack of any such pattern in our results implies that asymmetrical gaits may be confined to Crocodylia, that they evolved more than once in this lineage, or that our methods were not able to identify vertebral features associated with these gaits. Incorporation of other axial structures (e.g. osteoderms; soft tissues) into the models might change these results.

Maximum dorsoventral RoM (9–11° per joint in *Terrestrisuchus* and 15–21° in *Protosuchus*; table 4) certainly does not preclude the use of mammal-like asymmetrical gaits in early crocodylomorphs. In a range of small mammals across different asymmetrical gaits, maximum dorsoventral flexion (mostly produced by the last seven pre-sacral vertebrae) was only 40–51° [69]. However, osteological RoM is probably much larger than flexibility of the intact trunk (i.e. ligaments, muscles and skin prevent animals from using their full osteological RoM), and dorsoventral RoM was far smaller in *Terrestrisuchus* than in *Crocodylus* (9–11° versus 12–28°), which use asymmetrical gaits infrequently [30,70] and at slower speeds than cursorial mammals [29]. Therefore, early crocodylomorphs probably employed less dorsoventral movement (and resultant increase in step length and speed) than many extant mammals that use these types of gaits. This result reinforces our point [20] that, despite their convergent evolution of asymmetrical gaits, mammals and crocodylomorphs display key differences in vertebral function—and thus locomotor dynamics.

#### 4.2.2 Swimming

In many secondarily aquatic taxa, including other marine reptiles and cetaceans, adaptation to aquatic locomotion seems to have involved an initial decrease in stiffness of the vertebral column followed by a subsequent increase in stiffness. In cetaceans, for example, the initial decrease in stiffness is associated with adaptation for undulatory swimming with slow, high-amplitude waves of flexion followed by increase in stiffness (by different structural mechanisms) with adaptation for carangiform swimming using faster waves of smaller amplitude [71]. Our morphometric data support a similar sequence of changes in thalattosuchians (figure 7): in the semi-aquatic *Pelagosaurus*, relatively low mediolateral stiffness could indicate a similar locomotor pattern to basilosaurids (stem cetaceans), which swam by undulation of the lumbar and caudal regions [72], or simply a swimming style similar to extant crocodylians [24], which have a large mediolateral RoM but use axial swimming—in which the limbs are adducted, the trunk remains fairly straight, and thrust is produced by undulation of the tail—and, less often, paraxial swimming, in which the trunk also undulates [73]. *Crocodylus* would be analogous to amphibious archaeocetes (e.g. Ambulocetidae), which retained the stiffer lumbar vertebrae of their artiodactyl ancestors [71].

Morphometrics of the aquatic specialist *Metriorhynchus* are consistent with high mediolateral IVJ stiffness (relatively long vertebral processes implying large axial muscles; tall and fairly broad centra correlated with high IVJ stiffness in *Crocodylus* [20]), particularly in the cranial portion of the trunk, corresponding to higher frequency oscillations with amplitudes that increase towards the tail, similar to most modern whales [16]. The estimated mediolateral IVJ stiffness of *Steneosaurus* was intermediate between that of *Pelagosaurus* and *Metriorhynchus*, consistent with its reconstruction as an ambush predator rather than a pursuit predator like *Metriorhynchus*. Similar patterns of increase in vertebral stiffness with adaptation for rapid swimming have been inferred for secondarily aquatic marine reptiles, including ichthyosaurs [31], nothosaurs [34] and mosasaurs [32,33]. Thus, together, the available evidence on the evolution of vertebral form and function in aquatic tetrapods indicates widespread convergent evolution owing to common biomechanical principles and constraints.

### 4.3 Effects of other axial tissues

The contention of Salisbury & Frey [21] that axial mechanics depend more upon IVJs in modern crocodylians and more upon dorsal osteoderms in early crocodylomorphs provides a possible explanation for our unexpected results for *Terrestrisuchus* and *Protosuchus.* Our models showed relatively large mediolateral RoM in the thoracic vertebrae of *Protosuchus* (16–19°; figure 4 and table 4). However, if the paravertebral shield in early crocodylomorphs limited lateral and ventral flexion to 5–10° per joint, as Salisbury & Frey [21] predicted, they would have had the smallest mediolateral RoM of the studied taxa, consistent with primarily dorsoventral axial movements. Likewise, with a substantial increase in mediolateral IVJ stiffness incurred by the paravertebral shield in *Protosuchus* and *Terrestrisuchus*, the early crocodylomorphs would have greater mediolateral than dorsoventral stiffness, while the reverse would be true of the thalattosuchians and modern crocodylians, consistent with presumed locomotor behaviour in the extinct taxa. The ‘open’ paravertebral shield of *Pelagosaurus* is thought to have allowed greater lateral flexion than the ‘closed’ paravertebral shields of *Terrestrisuchus* and *Protosuchus*, but it may have restricted ventral flexion [21]. Combined with limited dorsal IVJ extension caused by the cranio-caudally elongate neural spines (table 4), this restriction would probably result in a smaller dorsoventral RoM in *Pelagosaurus* compared with the early crocodylomorphs, consistent with its more sprawling posture and aquatic habits. This hypothesis implies a functional trade-off between mobility and stiffness: with the use of more parasagittal kinematics in early crocodylomorphs, lateral movements of the vertebral column may have become less important than passive stiffness, and the axial column was stiffened via the paravertebral shield. In contrast, modern crocodylians, which use lateral undulation in terrestrial locomotion, have less rigid osteodermal armour and increase stiffness via large epaxial muscles and stiff IVJs (figure 7). The development of procoelous vertebrae in the recent ancestors of modern crocodylians also may have played a role in shifting the burden of gravitational support from the osteoderms to the IVJs [21], resulting in higher joint stiffness but greater RoM (figure 7). Our results showed that *Crocodylus* had substantially greater space between adjacent centra than the other taxa we studied (table 1), suggesting that the shift to procoelous IVJs may have involved an increase in the amount of soft tissue within the joint, increasing RoM. Future studies should investigate this speculation with a larger dataset.

By sequentially removing axial tissues in *Crocodylus* and measuring deflection and stiffness, we found that the most important structures for determining passive stiffness and trunk deflection varied between bending directions. In ventral flexion, IVJs were of primary importance; in dorsal extension, ribs and body wall musculature contributed to stiffness and osteoderms restricted trunk deflection. In contrast, in lateral flexion, ribs contributed to stiffness and each of the other structures restricted trunk deflection to some degree. Therefore, passive stiffness and RoM in ventral flexion in crocodylians presumably can be predicted based upon vertebral morphology alone. However, we did not test the effects of epaxial muscle activation, which probably resists ventral flexion in living crocodylians [21]. Our results emphasize that the effects of soft tissues and dermal ossifications should be considered when attempting to infer axial mechanics of the intact trunk, not just in terms of total RoM and stiffness, but also relative RoM and stiffness between bending directions.

### 4.4 Validity and accuracy of results

Estimates of stiffness and RoM in extinct animals should be approached with caution because mechanical properties of joints may be influenced by many factors that cannot be observed in fossils. However, our validation test of the RoM estimation method used in this study (figure 5) showed that differences in RoM between bending directions and major patterns of variation along the column could be predicted with confidence in *Crocodylus*. Of course, estimated RoM magnitudes are almost certainly larger than RoM in the living animal (or even isolated joints, such as those used for the validation test), and a greater sample size would be needed to predict the amount of overestimation. Our estimates of stiffness relied upon correlations with morphometric measurements found in previous studies, particularly Molnar *et al.* [20] because it is the only such study on non-mammalian tetrapods. However, it is possible that some of the relationships we had found are unique to extant crocodylians; similar studies on other related animals are required to test this possibility.

**Figure 9.**
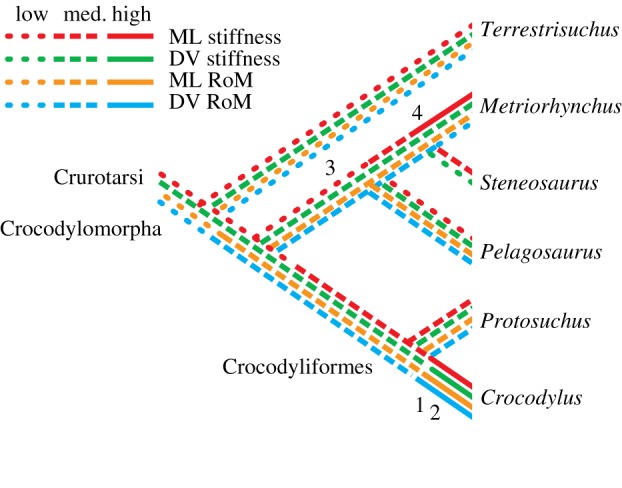
Hypothetical changes in IVJ stiffness and RoM with aquatic adaptation in Crocodylomorpha based on alternative phylogeny [76]; contrast with figure 7. Numbers show changes in other axial tissues: (1) sagittal segmentation of the paravertebral shield; (2) procoelous vertebrae; (3) ‘open’ margins of the paravertebral shield; (4) complete loss of osteoderms [21]. NB: procoelous vertebrae and/or loss of osteoderms evolved convergently in *Junggarsuchus* [7] and a few other crocodylomorph clades as well ([53] and references therein). ML, mediolateral; DV, dorsoventral.

Some of our hypotheses concerning the evolution of vertebral function are sensitive to the phylogenetic placement of Thalattosuchia within Crocodylomorpha. Although several recent analyses support the position of Thalattosuchia within Mesoeucrocodylia (e.g. [74,75]), at least one places Thalattosuchia outside of Crocodyliformes [76], which if correct would mean that low mediolateral stiffness in *Pelagosaurus*might represent retention of the ancestral condition rather than a specialization related to aquatic locomotion (figure 9).

## 5. Conclusion

Joint stiffness in mediolateral flexion tended to decrease with adaptation to aquatic locomotion in thalattosuchians, but the trend seems to have reversed somewhat in the aquatic specialist *Metriorhynchus*. IVJs of early crocodylomorphs were probably less stiff (based on morphometric correlates) but had smaller osteological RoM (based on virtual models) compared with those of modern crocodylians, in accord with the idea that osteoderms played a greater role in supporting the trunk in early crocodylomorphs [21]. Tissues other than vertebrae substantially influence the stiffness and RoM of the intact crocodylian trunk, and these effects vary across bending directions, so the effects of skin, osteoderms and other tissues should be taken into account when attempting to reconstruct the locomotion of extinct crocodylomorphs.

## Data accessibility

Non-normalized morphometric measurements have been included as part of the electronic supplementary material. Processed CT/micro-CT scan data in the form of STL files will be freely available from the 3D file repository M3 (http://morphomuseum.com): doi:10.18563/m3.1.3.e5. Raw scan data may be requested from the museums that house each specimen.
